# Severe Hypocalcemia and Hypomagnesemia with Denosumab in Advanced Chronic Kidney Disease: Case Report and Literature Review

**DOI:** 10.1155/2018/2059364

**Published:** 2018-10-14

**Authors:** Christina F. Marlow, Shailendra Sharma, Faizan Babar, Jianqing Lin

**Affiliations:** ^1^Department of Internal Medicine, George Washington University School of Medicine, USA; ^2^Division of Kidney Diseases and Hypertension, George Washington University School of Medicine, USA; ^3^Division of Hematology and Oncology, George Washington University School of Medicine, USA

## Abstract

**Background:**

Denosumab has become the preferred agent over zolendronic acid to help prevent skeletal-related events in patients with metastatic bone disease and multiple myeloma because it is approved for use in those with kidney dysfunction. However, denosumab has been linked to cases of hypocalcemia, particularly in those with advanced kidney disease.

**Case Presentation:**

We present the case of a patient with metastatic prostate cancer and chronic kidney disease due to obstructive nephropathy who developed severe hypocalcemia and hypomagnesemia after denosumab injection, which required intensive care unit admission, aggressive calcium supplementation, and hemodialysis assistance. We reviewed the evidence behind the safety profile of denosumab in chronic kidney disease, and we also looked at additional factors that may precipitate severe hypocalcemia with denosumab in advanced kidney disease.

**Conclusion:**

We believe that denosumab should be avoided in advanced chronic kidney disease due to the potential life-threatening, severe hypocalcemia that has been observed.

## 1. Introduction

Skeletal-related events (SREs) are a significant source of morbidity in patients with metastatic bone disease due to solid tumor malignancies and multiple myeloma. SREs are typically defined as pathologic fracture, spinal cord compression, need for bone radiation, or surgical intervention to bone [1]. SREs are associated with a significant decrease in quality of life and performance status. Zoledronic acid and denosumab are typically used to reduce SREs. Zoledronic acid is a bisphosphonate and denosumab is a monoclonal antibody that inhibits osteoclast-mediated bone resorption by binding to receptor activator of nuclear factor-*κ*B ligand (RANKL). In 2011, a randomized controlled trial conducted by Fizazi et al. showed that denosumab significantly delayed time to first SRE when compared to zoledronic acid (20.7 months versus 17.1 months, *p* = 0.0002) [2]. In terms of adverse side effects, bisphosphonates have been associated with renal toxicity and are contraindicated in patients with CrCl < 30 ml/min. Denosumab has emerged as an alternative agent in prevention of SREs in metastatic bone disease. However, denosumab is associated with a greater incidence of hypocalcemia [2]. There have been a number of cases reporting mild hypocalcemia associated with denosumab use [3, 4]. We present the case of a patient with metastatic prostate cancer who developed severe hypocalcemia in the setting of advanced chronic kidney disease (CKD) after receiving denosumab. We also searched the literature to evaluate the safety of denosumab use in chronic kidney disease.

## 2. Case

A 70-year-old African American gentleman with a history of CKD, pseudogout, and metastatic castration-resistant prostate cancer (to bone, nodes, and lung) was admitted to the hospital due to left knee swelling. He was incidentally found to be severely hypocalcemic to 2.7 mg/dl with EKG showing a prolonged QTC interval to 525 ms. Physical examination was negative for Chvostek and Trousseau's sign. Home medications included acetaminophen, amlodipine, bicalutamide, docusate-senna, lidocaine patches, ondansetron, and polyethylene glycol. Twenty-eight days prior, he had received his first dose of denosumab (together with leuprolide) when his calcium level was 8.8 mg/dl. He was prescribed vitamin D and calcium supplementation, but never took it. He finished a five-day course of prednisone 10 mg daily thirteen days prior for a pseudogout flare of the right foot. Other labs on admission were significant for phosphorus 5.5 mg/dl, total vitamin D3 31 IU, iPTH 93, and Mg 1.1 mg/dl. He was admitted to the intensive care unit for continuous, high-dose IV calcium gluconate and frequent electrolyte monitoring. During his ICU course, he received a total of 21 grams of IV calcium gluconate. He was also given 1 *μ*g of oral calcitriol BID, 1000 mg of oral calcium carbonate TID, and aggressive magnesium supplementation. Unfortunately, the patient's creatinine worsened to 7.5 mg and he developed severe metabolic acidosis and hyponatremia in the setting of *Clostridium difficile* colitis. The decision was made to initiate hemodialysis given the poor recovery in kidney function from obstructive uropathy. For one week, he received intermittent hemodialysis with high calcium baths. Calcium, magnesium, and phosphate levels were monitored daily and supplemented as needed. Calcium levels improved to 8.5 mg/dl and magnesium levels improved to 2.0 mg/dl. He was discharged on 0.5 *μ*g oral calcitriol daily, calcium carbonate 1000 mg BID, and continued intermittent hemodialysis.

The patient was originally diagnosed with de novo metastatic prostate cancer two years prior. He had not been on ADT or chemotherapy for approximately one year until he presented to our hospital two months prior with evidence of prostate cancer progression. His PSA was elevated to 1472 and he was found to have acute kidney injury with a Cr 12.8 mg/dl and a BUN of 131 mg/dl due to obstructive uropathy. CT of the abdomen/pelvis showed marked bilateral hydronephrosis due to a 6.3 cm pelvic mass and a 3.8 cm mass in the left ureteropelvic junction. Extensive pelvic and retroperitoneal lymphadenopathy was evident in addition to innumerable metastatic lytic skeletal lesions. Bilateral percutaneous nephrostomy tubes were placed by interventional radiology and the patient's creatinine improved to 7.3 mg/dl with a BUN of 120 mg/dl. Combined androgen deprivation was resumed and he was discharged with plans for close monitoring of renal functions with the ultimate plan to place ureteral stents depending upon renal recovery.

## 3. Discussion

Denosumab is a RANKL monoclonal antibody that blocks the RANKL receptor on the osteoclast, which results in reduced osteoclast activation and resultant bone resorption. Denosumab is more potent compared to bisphosphonates in preventing skeletal-related events in metastatic prostate cancer [2]. However, compared to bisphosphonates, the incidence of hypocalcemia is higher in patients treated with denosumab [2]. Although denosumab does not require renal dosing, the manufacturer recommends extreme caution with close monitoring of calcium levels in chronic kidney disease [5]. The risk of hypocalcemia with denosumab increases in patients with advanced stage kidney disease due to inherent defects in bone mineral metabolism and dependence on PTH-mediated bone resorption to maintain calcium levels.

Knowledge of the pharmacokinetics of denosumab is critical in understanding the risk of hypocalcemia. Denosumab exhibits a nonlinear dose-dependent response [6, 7]. The volume of distribution is proportional to the body weight and central volume of distribution is 2.6 l/66 kg body weight [6, 7]. Maximum drug level is reached in seven to twenty-one days. The drug elimination is not dependent on renal or hepatic clearance [6, 7]. Hence, no dose adjustment is recommended for patients with CKD. Like other monoclonal antibodies, clearance is mediated by the reticuloendothelial system and receptor-mediated endocytosis [6, 7]. Given the long half-life of the medication, side effects may persist for a prolonged period.

Denosumab is preferred over zoledronic acid in patients with both metastatic bone disease and chronic kidney disease because it is not cleared renally and is technically not nephrotoxic. However, there is a paucity of data regarding the safety of denosumab use in patients with advanced renal disease, that is, GFR < 30 ml/min or on hemodialysis, for the prevention of skeletal-related events in metastatic bone disease. An abstract of an open-label prospective study describes the effectiveness of denosumab at different stages of CKD in preventing SREs at the standard dose [8, 9]. However, severe hypocalcemia (i.e., <7 meq/dl) or symptomatic hypocalcemia was seen in the late stages of renal disease [10–12]. Similarly, in one observational study, 45 percent of the patients with baseline eGFR < 30 ml/min (*n* = 22) developed hypocalcemia during treatment with denosumab [13]. A dose-reduction strategy in CKD has been evaluated in only one case series study by Cicci et al. of refractory hypercalcemia due to MM and AKI [14]. They proposed the novel dosing strategy of 0.3 mg/kg of denosumab based on 4 case reports in patients with multiple myeloma over a fixed, reduced dose of 60 mg (from the standard 120 mg) in patients with renal dysfunction. Denosumab was used to treat refractory hypercalcemia associated with MM. Despite dose reduction, three out of four patients developed mild hypocalcemia with the effect lasting for 15–40 days [14]. Dose reduction might be an effective strategy to reduce the risk of severe hypocalcemia in CKD, but it requires further validation in large scale studies.

**Figure 1:**
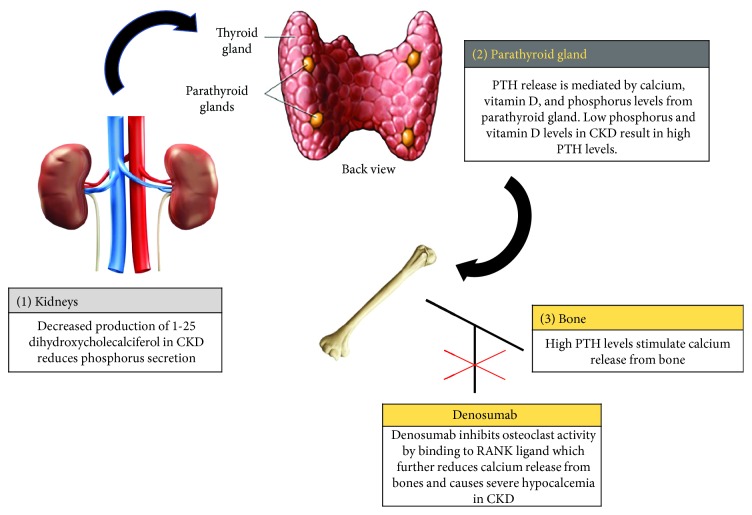
Calcium, phosphorus, and vitamin D metabolism in chronic kidney disease.

The higher risk of severe hypocalcemia in advanced kidney disease is due to the secondary hyperparathyroidism that is known to occur in end stage chronic kidney disease (Figure 1). Denosumab then further compounds the risk for hypocalcemia by blocking calcium release from the bone. In two trials evaluating the safety and efficacy of denosumab in patients with CKD stages 4 and 5, all patients received supplementation with calcium and vitamin D [5]. Despite these measures, patients developed hypocalcemia of variable degrees [13]. Hence, we believe that supplementing a vitamin D analogue prior to denosumab use in advanced CKD is unlikely to be an effective way of reducing hypocalcemia risk.

We also identified additional factors that may predispose advanced kidney disease patients to severe hypocalcemia with denosumab use including high bone turnover markers, hypomagnesemia, and concomitant use of steroids or bisphosphonates. Bone turnover markers can be helpful in predicting patients at risk for hypocalcemia because high bone turnover is seen with both bone mineral disease and metastatic bone disease [15]. Several markers have been shown to correlate with bone activity in patients with CKD who were treated with denosumab for osteoporosis including bone specific alkaline phosphatase, CTX, total P1NP, and TRAP [15–17]. Further studies should explore the utility of checking these markers prior to administration of denosumab to those at greater risk of severe hypocalcemia.

Hypomagnesemia is another risk factor that can predispose to severe hypocalcemia with denosumab use in CKD which was evident in the case of this patient. Low magnesium is observed in chronic kidney disease patients with diabetes [18]. Magnesium acts as a cofactor to calcium-sensing receptors on the parathyroid gland to regulate PTH secretion [19]. Low Mg levels increase PTH release, which increases bone turnover. Hypomagnesemia is also associated with low calcitriol levels which can also increase the risk of hypocalcemia with denosumab use in CKD [20]. Additional risk factors for severe hypocalcemia with denosumab use include concomitant use of hypocalcemia agents such as steroids, bisphosphonates, and calcimimetics [13]. This was particularly evident in this case as the patient received five days of steroids for pseudogout prior to the first dose of denosumab.

## 4. Conclusion

Denosumab is an effective agent at reducing SREs in patients with metastatic bone disease and is technically approved for use in CKD patients without dose reduction. Inherent bone mineral disease in advanced kidney disease increases the risk of hypocalcemia with denosumab use. Prospective studies are needed to explore the safety profile of denosumab at a reduced dose in CKD patients. Further research is needed to determine the clinical utility of bone turnover markers; however, they may be helpful tools to physicians to help determine a patient's risk of hypocalcemia prior to starting denosumab. Until further evidence, we believe that denosumab should be avoided in advanced chronic kidney disease due to the potential life-threatening, severe hypocalcemia that has been observed.
